# Assessing the HIV Care Continuum in Latin America: progress in clinical retention, cART use and viral suppression

**DOI:** 10.7448/IAS.19.1.20636

**Published:** 2016-04-08

**Authors:** Peter F Rebeiro, Carina Cesar, Bryan E Shepherd, Raquel B De Boni, Claudia P Cortés, Fernanda Rodriguez, Pablo Belaunzarán-Zamudio, Jean W Pape, Denis Padgett, Daniel Hoces, Catherine C McGowan, Pedro Cahn

**Affiliations:** 1 Department of Medicine, Division of Infectious Diseases, Vanderbilt University School of Medicine, Nashville, TN, USA; 2 Fundación Huésped, Buenos Aires, Argentina; 3 Instituto Nacional de Infectologia Evandro Chagas, Fundação Oswaldo Cruz, Rio de Janeiro, Brazil; 4 Fundación Arriarán, Universidad de Chile, Santiago, Chile; 5 Departamento de Infectología, Instituto Nacional de Ciencias Médicas y Nutrición Salvador Zubirán, Mexico City, Mexico; 6 Le Groupe Haïtien d'Etude du Sarcome de Kaposi et des Infections Opportunistes (GHESKIO), Port-au-Prince, Haiti; 7 Instituto Hondureño de Seguridad Social and Hospital Escuela, Tegucigalpa, Honduras; 8 Instituto de Medicina Tropical Alexander von Humboldt, Universidad Peruana Cayetano Heredia, Lima, Peru

**Keywords:** HIV Care Continuum, Latin America, retention, cART use, viral suppression, cohort studies

## Abstract

**Introduction:**

We assessed trends in HIV Care Continuum outcomes associated with delayed disease progression and reduced transmission within a large Latin American cohort over a decade: clinical retention, combination antiretroviral therapy (cART) use and viral suppression (VS).

**Methods:**

Adults from Caribbean, Central and South America network for HIV epidemiology clinical cohorts in seven countries contributed data between 2003 and 2012. Retention was defined as two or more HIV care visits annually, >90 days apart. cART was defined as prescription of three or more antiretroviral agents annually. VS was defined as HIV-1 RNA <200 copies/mL at last measurement annually. cART and VS denominators were subjects with at least one visit annually. Multivariable modified Poisson regression was used to assess temporal trends and examine associations between age, sex, HIV transmission mode, cohort, calendar year and time in care.

**Results:**

Among 18,799 individuals in retention analyses, 14,380 in cART analyses and 13,330 in VS analyses, differences existed between those meeting indicator definitions versus those not by most characteristics. Retention, cART and VS significantly improved from 2003 to 2012 (63 to 77%, 74 to 91% and 53 to 82%, respectively; *p*<0.05, each). Female sex (risk ratio (RR)=0.97 vs. males) and injection drug use as HIV transmission mode (RR=0.83 vs. male sexual contact with males (MSM)) were significantly associated with lower retention, but unrelated with cART or VS. MSM (RR=0.96) significantly decreased the probability of cART compared with heterosexual transmission.

**Conclusions:**

HIV Care Continuum outcomes improved over time in Latin America, though disparities for vulnerable groups remain. Efforts must be made to increase retention, cART and VS, while engaging in additional research to sustain progress in these settings.

## Introduction

The HIV *cascade of care* or *care continuum* is a powerful epidemiologic and programmatic framework describing the movement of HIV-positive individuals through discrete stages of care, from diagnosis and linkage to care, to clinical retention, combination antiretroviral therapy (cART) use and ultimately viral suppression (VS). There are numerous advantages to conceptualizing the clinical experiences of persons living with HIV/AIDS in this manner, chief among them the ability to identify specific and simply defined programmatic or clinical actions that, if improved, would entail improved HIV outcomes in a treatment as prevention paradigm [1]. Though challenges in data acquisition and measurement accuracy for these indicators remain, perhaps differentially depending on the setting, the implementation of care continuum assessments in low-, middle- and high-income countries is critical to track progress toward the goals of increased successful participation in care at each stage [2]. Progress across the continuum is necessary to improve individual-level HIV disease outcomes and reduce population-level transmission, and related goals have been enunciated by the World Health Organization as part of its revised 2013 cART guidelines [3].

In addition to expanding the scope of the descriptive epidemiology of HIV, the care continuum has also come to be recognized as a useful tool for assessing progress in the expansion of cART programmes, highlighting disparities in disease progression among key populations and in identifying points of intervention to improve treatment outcomes in adult populations in Asia, Europe, North America and sub-Saharan Africa [4–10]. This critical assessment of programme implementation and treatment targets through the lens of the care continuum offers epidemiologists and policymakers insight into needed alterations to programmatic conduct and helps inform programme planning for future work, transforming evidence into public health action.

Thus far, however, no longitudinal application of this framework has been attempted across national boundaries in Latin America. Though changes in access to healthcare and cART occurred due to changing national policies and treatment guidelines across this region, populations of concern may not have experienced their benefits uniformly. Despite potential social and political barriers to care, though, many experienced functionally universal access to health services through public pension and single-payer government programmes or charitably funded clinical centres [11–14]. Given the non-trivial disease burden in the region, which accounts for 6% of global infections, we assessed trends in three care continuum indicators within the large and diverse Caribbean, Central and South America network for HIV epidemiology (CCASAnet) cohort over 10 years of follow-up [14].

## Methods

### Study population, follow-up and study outcomes

CCASAnet is a collaborative HIV cohort including sites in seven countries, and it is one of the seven member regions of the NIH-funded International Epidemiologic Databases to Evaluate AIDS (IeDEA) consortium [14]. CCASAnet sites contributing data to this study were Hospital Fernandez and Centro Médico Huésped, Buenos Aires, Argentina (HF/CMH-Argentina); Instituto Nacional de Infectologia Evandro Chagas, Fundação Oswaldo Cruz, Rio de Janeiro, Brazil (FC-Brazil); Fundación Arriarán, Santiago, Chile (FA-Chile); Le Groupe Haïtien d'Etude du Sarcome de Kaposi et des Infections Opportunistes, Port-au-Prince, Haiti (GHESKIO-Haiti); Instituto Hondureño de Seguridad Social and Hospital Escuela, Tegucigalpa, Honduras (IHSS/HE Honduras); Instituto Nacional de Ciencias Médicas y Nutrición Salvador Zubirán, Mexico City, Mexico (INCMNSZ-Mexico); and Instituto de Medicina Tropical Alexander von Humboldt, Lima, Peru (IMTAvH-Peru). Clinical and epidemiological data were collected at each site, de-identified and sent to the CCASAnet Data Coordinating Center at Vanderbilt University (VDCC; Nashville, TN, USA), for data harmonization. Data quality checks and on-site audits were performed by VDCC to ensure data accuracy. Institutional review board approval was obtained from each site and from Vanderbilt University.

Adults (≥18 years old) participating in these cohorts were included if they contributed clinical visit or laboratory data from their first visit between 2003 and 2012 and were present in the cohort for more than one year; they potentially contributed multiple visits across all 10 years. Sites in Argentina and Peru did not contribute visit dates, and dates of laboratory measures of CD4+ lymphocyte count (CD4) or HIV-1 RNA viral load (VL) were therefore used as proxies for visits [15].

HIV Care Continuum indicators of clinical retention, use of cART and VS were the outcomes of interest.

Retention was defined using the US Institute of Medicine retention indicator: at least two HIV care visits in a year, >90 days apart [15]. Patients were included in the retention denominator in every calendar year after their year of cohort entry until the final recorded visit, death or the end of 2012. Exclusions from the denominator occurred due to individuals not being uniformly at risk for the outcome during the entire year. For example, patients who died during the first quarter of a calendar period could not possibly be at risk for being retained in that year, and no patient would be at risk for retention subsequent to death in that calendar period, so patients did not contribute observations in the year of death.

cART use was defined as prescription of at least one regimen of at least three active antiretroviral agents in a year [16]. Though cART adherence was not measured and influences the VS indicator, we believe that cART prescription is an indicator of programmatic success and an upper bound on treatment success for that particular stage of the care continuum. Patients from GHESKIO Haiti were excluded from analyses for cART use, because this site only sent data from patients who started cART.

VS was an HIV-1 RNA VL<200 copies/mL at the last measurement in the year [16]. The lower limit of quantitation for some VL assays used during the study period was 400 copies/mL, and because only 5% of VLs in this study population were at this limit of detection, these VLs were also designated as meeting the requirements for VS. The resultant misclassification error was likely minimal as evidenced by the fact that only 2% of VLs measured using assays known to have a 200-copy limit of detection were between 200 and 400 copies/mL. Patients from GHESKIO Haiti were excluded from analyses for VS because VLs were not routinely performed. Patients were excluded from the cART use and VS denominators if they did not complete at least one visit or, for Argentinian and Peruvian sites, at least one laboratory measure in each calendar year. Patients were excluded from the VS denominator if they did not obtain a VL measurement in that year. As VS was the primary outcome for the VS indicator analyses, missing VL data were not imputed. The cART use and VS outcomes were not conditioned on patients being retained in care (according to the first outcome defined), though these outcomes were measured among patients receiving care. Patients were also excluded from analyses for any indicator if there were fewer than 100 patients contributing at that site in any calendar period (e.g. IHSS/HE Honduras pre-2007 for VS) (Supplementary Figure 1).

**Figure 1:**
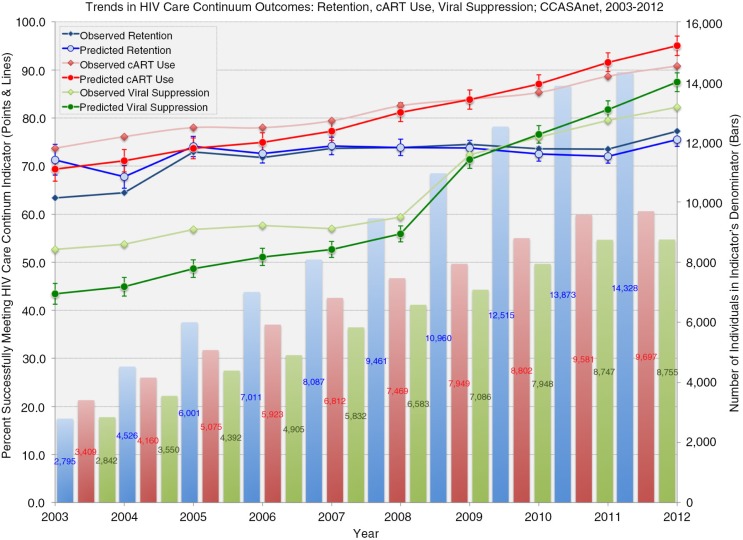
Temporal trends in observed and predicted* HIV Care Continuum indicators among individuals in the Caribbean, Central and South America network for HIV epidemiology (CCASAnet), from 2003 through 2012. Denominator bars are presented in the same colours as observed and predicted percentages meeting indicator definitions in each year: blue for retention, red for cART use, and green for viral suppression. *Predicted percentages and 95% confidence intervals are derived from multivariable modified Poisson regression models using a generalized estimating equation (GEE) to account for within-individual correlation of multiple outcomes and either unstructured (retention and viral suppression analyses) or exchangeable (cART analysis) correlation structures. All models were adjusted for age, sex, HIV risk factor, contributing cohort site, calendar year, and total time in care. Age and calendar period were modelled using restricted cubic splines with four knots.

Individual clinical visit, laboratory and cART data were used to create one observation per year between the year of entry into the cohort and the year of a final visit or laboratory measurement prior to the end of 2012. This single annual observation carried a summary of the individual's retention status, cART use during the year or VS status, depending on whether the individual's data for that year met criteria for those outcomes. Follow-up time ranged between a minimum of one and a maximum of 10 years.

### Factors investigated for association with outcomes

Patient age each year, sex, likely HIV transmission category (men who have sex with men (MSM), injection drug use (IDU), heterosexual contact, or unknown or other risk), country of care and calendar year were investigated for associations with the outcomes and to assess trends over time. Total individual time contributed to the study and cohort site (Argentina and Honduras had two sites) were used as adjustment factors for all models.

### Statistical analysis

Differences in outcomes by patient characteristics were assessed using bivariate modified Poisson regression models, accounting for multiple outcomes per individual. Multivariable modified Poisson regression models were used to assess risk ratios (RRs) and 95% confidence intervals (CIs) for associations between patient characteristics and the outcomes, as well as to assess temporal trends and predict percentages meeting each indicator in each year, adjusting for age, sex, likely HIV transmission category, cohort site (within country), calendar year and total time in care [17]. All regression models used a generalized estimating equation (GEE) to account for within-individual correlation of multiple outcomes and either unstructured (retention and VS analyses) or exchangeable (cART analysis) correlation structures [18]. Predicted values for each outcome were the predictive margins: the modelled predictive values of each outcome for each patient each year, averaged over all patients in the study. Age and calendar year were modelled using restricted cubic splines with four knots [19]. For calendar year, knots were at 2004, 2008, 2010 and 2012. All analyses were conducted in Stata 12.1 (StataCorp, College Station, TX, USA).

## Results

Among 18,799 individuals contributing 89,557 person-years to retention analyses; 14,380 contributing 68,877 person-years to cART use analyses; and 13,330 contributing 60,640 person-years to VS analyses, there were significant improvements in each of the indicators from 2003 to 2012: from 63 to 77% retained, 74 to 91% using cART and 54 to 83% virally suppressed (*p*<0.05, each). Disparities between retention and the other indicators were due to retention operating independently of the other indicators, relying solely on clinic attendance and not conditioned on as a prerequisite for the other indicators. Covariate-adjusted estimates from the full models revealed similar trends over calendar time (Figure 1). There were, however, differences between those meeting indicator definitions and those not by most characteristics (Table 1). Median age differed significantly and consistently by each outcome, with slightly higher median ages comparing those retained to not retained (36.4 vs. 35.2 years), on cART to those not on cART (35.5 vs. 32.5 years) and those with VS to those without VS (35.9 vs. 33.4 years). Outcomes also varied across countries, with retention ranging between 59.8% in Argentina and 85.1% in Honduras, cART use ranging between 81.0% in Argentina and 92.2% in Honduras, and VS ranging between 62.7% in Peru and 87.4% in Honduras.

**Table 1:** Characteristics of individuals in the Caribbean, Central and South America network for HIV epidemiology (CCASAnet) contributing to analyses of trends in HIV Care Continuum indicators from 2003 through 2012: retention, cART use, and viral suppression

Characteristic	Total for retention^a^ (*N*=18,799)	Not retained^a^	Retained^a^	p*	Total for cART^b^ (*N*=14,380)	Not on cART^b^	On cART^b^	p*	Total for viral suppression^c^ (*N*=13,330)	Not virally suppressed^c^	Virally suppressed^c^	p*
Total	89,557	23,869	65,688		68,877	11,565	57,312		60,640	17,708	42,932	
Age (years)	36.1 (30.0, 43.0)	35.2 (29.3, 41.7)	36.4 (30.3, 43.5)	0.21	35.0 (29.1, 41.9)	32.5 (27.1, 39.3)	35.5 (29.6, 42.4)	<0.01	35.3 (29.3, 42.1)	33.4 (27.6, 40.2)	35.9 (30.0, 42.9)	<0.01
Sex												
Male	56,725	15,321 (27.0)	41,404 (73.0)	Ref.	49,101	8119 (16.5)	40,982 (83.5)	Ref.	43,474	12,357 (28.4)	31,117 (71.6)	Ref.
Female	32,832	8548 (26.0)	24,284 (74.0)	0.24	19,776	3446 (17.4)	16,330 (82.6)	0.70	17,166	5351 (31.2)	11,815 (68.8)	0.11
HIV risk factor												
MSM	25,553	7515 (29.4)	18,038 (70.6)	Ref.	27,304	5079 (18.6)	22,225 (81.4)	Ref.	24,026	6970 (29.0)	17,056 (71.0)	Ref.
IDU	1555	820 (52.7)	735 (47.3)	<0.01	1344	203 (15.1)	1141 (84.9)	0.25	1191	340 (28.6)	851 (71.5)	0.96
Hetero	28,938	9454 (32.7)	19,484 (67.3)	<0.01	29,745	4800 (16.1)	24,945 (83.9)	0.17	25,965	7988 (30.8)	17,977 (69.2)	0.56
Other/unknown	33,511	6080 (18.1)	27,431 (81.9)	<0.01	10,484	1483 (14.2)	9001 (85.9)	<0.01	9458	2410 (25.5)	7048 (74.5)	<0.01
Country												
Argentina^d^	18,878	7598 (40.2)	11,280 (59.8)	Ref.	18,721	3549 (19.0)	15.172 (81.0)	Ref.	17,282	5127 (29.7)	12,155 (70.3)	Ref.
Brazil	17,399	4922 (28.3)	12,477 (71.7)	<0.01	18,318	3253 (17.8)	15,065 (82.2)	0.34	17,167	5215 (30.4)	11,952 (69.6)	0.78
Chile	11,938	2723 (22.8)	9215 (77.2)	<0.01	12,548	2282 (18.2)	10,266 (81.8)	0.80	10,116	2618 (25.9)	7498 (74.1)	0.20
Haiti^e^	4279	1027 (24.0)	3252 (76.0)	<0.01	N/A	N/A	N/A		N/A	N/A	N/A	
Honduras	23,074	3438 (14.9)	19,636 (85.1)	<0.01	3012	234 (7.8)	2778 (92.2)	<0.01	1324	167 (12.6)	1157 (87.4)	<0.01
Mexico	3933	593 (15.1)	3340 (84.9)	<0.01	4711	459 (9.7)	4252 (90.3)	<0.01	4556	778 (17.1)	3778 (82.9)	<0.01
Peru^d^	10,056	3568 (35.5)	6488 (64.5)	<0.01	11,567	1788 (15.5)	9779 (84.5)	<0.01	10,195	3803 (37.3)	6392 (62.7)	<0.01
Individual years in care	7 (4, 9)	7 (4, 9)	7 (4, 9)	<0.01	8 (5, 10)	6 (3, 8)	8 (5, 10)	<0.01	7 (5, 10)	6 (4, 9)	8 (5, 10)	<0.01

cART, combination antiretroviral therapy; Hetero, heterosexual contact; IDU, injection drug use; MSM, male sexual contact with men.

Numbers are presented as person-years contributed (%) except for age and individual years in care, which are median (interquartile range).

* Univariate modified Poisson regression with a generalized estimating equation (GEE) to account for within-individuals clustering of outcomes; *p*-value for age is for the Wald chi-square test when modelling age using a restricted cubic spline with four knots.

a US Institute of Medicine retention indicator: individuals with two or more HIV primary care encounters per year, >90 days apart

b cART was defined as regimens of three or more active antiretroviral agents (including triple-nucleoside regimens); US Department of Health and Human Services cART indicator: number of individuals prescribed cART during the year, among those with at least one HIV primary care visit during the year

c US Department of Health and Human Services viral suppression indicator: individuals with plasma HIV-1 RNA <200 copies/mL at the last measurement in the year, among those with at least one HIV primary care visit during the year

d Argentina and Peru used laboratory measures (CD4+ counts and HIV-1 RNA measures) as proxies for HIV primary care visits when determining retention status

e Haiti did not contribute to the assessment of cART use due to receipt of cART being an inclusion criterion of the clinical cohort; Haiti did not contribute to the assessment of viral suppression due to a lack of universal HIV-1 RNA testing within the clinical cohort.

In bivariate regression models for the retention outcome, IDU (RR=0.65; 95% CI: 0.58, 0.74) and heterosexual (RR=0.95; 95% CI: 0.92, 0.98) HIV transmission categories were associated with decreased probability of retention compared to MSM, whereas the HIV transmission category “other/unknown” (RR=1.16; 95% CI: 1.12, 1.19 vs. MSM) and younger age (RR=1.05; 95% CI: 1.00, 1.10 for 20 vs. 40 years) were associated with higher probability of retention. After adjustment in the full multivariable model, among men, IDU remained associated with reduced probability of retention (RR=0.83; 95% CI: 0.74, 0.93 vs. MSM), and among non-MSM, female sex (RR=0.97; 95% CI: 0.94, 0.99 vs. males) became associated with reduced probability of retention (Table 2).

**Table 2:** Modelled relationships between population characteristics of CCASAnet patients and HIV Care Continuum indicators from 2003 through 2012, with 95% confidence intervals: retention, cART use, and viral suppression

Characteristic	Unadjusted RR (95% CI): retention^a^	Adjusted* RR (95% CI): retention^a^	Unadjusted RR (95% CI): cART use^b^	Adjusted* RR (95% CI): cART use^b^	Unadjusted RR (95% CI): virally suppressed^c^	Adjusted* RR (95% CI): virally suppressed^c^
Age (years)^d^						
20	**1.05** (1.00, 1.10)	1.04 (0.99, 1.09)	**0.77** (0.73, 0.82)	**0.83** (0.78, 0.87)	**0.73** (0.69, 0.78)	**0.79** (0.75, 0.84)
30	1.00 (0.97, 1.02)	1.01 (0.98, 1.03)	**0.93** (0.90, 0.96)	**0.94** (0.91, 0.98)	**0.91** (0.88, 0.94)	**0.93** (0.90, 0.97)
40	Ref.	Ref.	Ref.	Ref.	Ref.	Ref.
50	1.01 (0.99, 1.03)	1.01 (1.00, 1.03)	**1.02** (1.00, 1.04)	**1.03** (1.01, 1.05)	**1.04** (1.02, 1.06)	**1.04** (1.02, 1.06)
60	1.02 (0.97, 1.07)	1.03 (0.98, 1.08)	**1.03** (0.97, 1.09)	**1.05** (0.99, 1.12)	**1.07** (1.01, 1.14)	**1.09** (1.03, 1.15)
Sex						
Male	Ref.	Ref.	Ref.	Ref.	Ref.	Ref.
Female	1.01 (0.99, 1.04)	**0.97** (0.94, 0.99)	0.99 (0.96, 1.03)	0.97 (0.93, 1.00)	0.97 (0.94, 1.01)	0.97 (0.94, 1.01)
HIV risk factor						
MSM	Ref.	Ref.	Ref.	Ref.	Ref.	Ref.
IDU	**0.65** (0.58, 0.74)	**0.83** (0.74, 0.93)	1.06 (0.96, 1.17)	1.09 (0.98, 1.20)	0.99 (0.89, 1.11)	1.03 (0.93, 1.15)
Hetero	**0.95** (0.92, 0.98)	1.00 (0.97, 1.04)	1.02 (0.99, 1.05)	**1.04** (1.01, 1.08)	0.99 (0.96, 1.02)	1.01 (0.97, 1.05)
Other/unknown	**1.16** (1.12, 1.19)	0.97 (0.93, 1.02)	**1.10** (1.05, 1.14)	1.05 (1.00, 1.10)	**1.10** (1.04, 1.14)	1.03 (0.97, 1.08)
Individual years in care	**1.01** (1.01, 1.01)	**1.02** (1.02, 1.02)	**1.05** (1.04, 1.05)	**1.05** (1.04, 1.05)	**1.05** (1.05, 1.06)	**1.06** (1.06, 1.07)

cART, combination antiretroviral therapy; CI, confidence interval; Hetero, heterosexual contact; IDU, injection drug use; MSM, male sexual contact with men; RR, risk ratio.

Bold estimates are statistically significant, *p*<0.05.

* Fully adjusted models include all terms in table, as well as cohort site and calendar time (modelled with a restricted cubic spline with four knots).

a US Institute of Medicine retention indicator: individuals with two or more HIV primary care encounters per year, >90 days apart; Argentina and Peru used laboratory measures (CD4+ counts and HIV-1 RNA measures) as proxies for HIV primary care visits when determining retention status

b cART was defined as regimens of three or more active antiretroviral agents (including triple-nucleoside regimens); US Department of Health and Human Services cART indicator: number of individuals prescribed cART during the year, among those with at least one HIV primary care visit during the year; Haiti did not contribute to the assessment of cART use due to receipt of cART being an inclusion criterion of the clinical cohort

c US Department of Health and Human Services viral suppression indicator: individuals with plasma HIV-1 RNA <200 copies/mL at the last measurement in the year, among those with at least one HIV primary care visit during the year; Haiti did not contribute to the assessment of viral suppression due to a lack of universal HIV-1 RNA testing within the clinical cohort

d Age was modelled using a restricted cubic spline with four knots; estimates presented are not age categories, but rather contrasting comparisons at specific age values (vs. the reference of 40 years old).

For the cART use outcome, in bivariate regression, older age was associated with increased probability of cART use (Table 2). Other/unknown HIV transmission category was associated with a higher probability of cART use versus MSM (RR=1.10; 95% CI: 1.05, 1.14). In the full multivariable model, age remained significantly associated with the same increasing dose-response relationship, and heterosexual HIV transmission category among males was associated with increased probability of cART use (RR=1.04; 95% CI: 1.01, 1.08 vs. MSM) (Table 2).

For the VS outcome, in bivariate regression, older age was associated with increased probability of VS, in an increasing dose-response fashion as with cART use (Table 2). Other/unknown HIV transmission category was associated with a higher probability of VS (RR=1.10; 95% CI: 1.05, 1.15). In the full multivariable model, only increasing age remained associated (in the same dose-response fashion) with increased probability of VS (Table 2).

Across all outcomes, increasing individual time in care was associated with increased probability of having each outcome (Table 2).

## Discussion

In assessing the three HIV Care Continuum outcomes of clinical retention, cART use and VS in the CCASAnet cohort over a decade, improvements were seen over time in these varied Latin American settings. cART use may have improved over time due to changes in ART guidelines and availability, and VS percentages may have improved due to the improved efficacy and tolerability of newer antiretroviral drugs, though these improvements were only measured among those receiving care. In particular, CD4 thresholds as treatment guidelines differed by clinical site and over time. Although the population meeting treatment guidelines was selected, the high proportion receiving cART in this group (>85% at all sites; >95% for many sites throughout most of the study) is a testament to how closely clinical guidelines were followed. However, these individuals comprised a relatively small proportion of the total population (15 to 25% during the study), and because the proportion receiving cART among those *not* meeting the CD4 threshold remained >70%, we believe our analysis remains relevant. It represents a retrospective assessment of cART receipt in a treatment as prevention context, whether or not that was the dominant paradigm at the time. Additionally, although provisions in law existed to protect marginalized groups and promote access to health services in several contributing countries during the study, we were not able to measure these and remain unsure of their particular contextual contribution to observed trends [14]. Even so, after accounting for potential cohort effects and other longitudinal changes, several demographic, clinical and likely HIV transmission category factors emerged as important predictors of suboptimal outcomes.

When examining the retention outcome, history of IDU as HIV transmission category among males and female sex were associated with a reduced probability of being retained. The association of poor retention with IDU was particularly troubling due to its magnitude and the proportion of more than 50% unretained. Though these associations were detected while accounting for all other measured factors, the impact of unmeasured determinants such as comorbid mental health issues, social stigma, lack of access to healthcare and lack of employment cannot be discounted. The IDU transmission category represents a very small fraction (<2%) of the HIV-infected population in this study, which is in accordance with the United Nations Office on Drugs and Crime data estimating a 0.33% point prevalence of IDU (among the general population aged 15 to 64) in Latin America and the Caribbean [20]. This figure is substantially lower than in Eastern/South-Eastern Europe (1.26%) or North America (0.66%). Although the prevalence of IDU may be lower in our setting, these data further demonstrate the importance of continued monitoring to improve retention in care and provide specific treatment needs to these patients [21]. Unlike similar outcomes observed in other settings, however, younger age was not a significant predictor of poor retention in care [22–24]. Whether due to other attendant risk behaviours, altered health-seeking behaviours or ongoing substance use, programmes providing substance use treatment and counselling, as well as interventions to promote more consistent care-seeking behaviour among females, particularly those of childbearing age, must be prioritized [5].

With respect to the probability of cART use, as seen in resource-rich countries, younger individuals were at heightened risk of suboptimal outcomes. Men with MSM as likely HIV transmission category were also at a slightly heightened risk of not being on cART. This is counter to what has been observed in multiple cohorts in resource-rich countries such as the United States and Canada, where MSM patients tend to have better retention, better use of cART and better proportions with VS than those in other transmission categories [22–24]. This observation is troubling because the HIV epidemic is concentrated among key, high-risk, high-burden populations (including MSM) in many settings globally, including Latin America. Lack of cART access and VS may jeopardize treatment as prevention strategies for a highly needy population. However, it should be noted that the adjusted reduced probability of receipt of cART was only 0.96 (vs. those with heterosexual transmission), and the unadjusted proportion receiving cART was more than 81% among MSM in this cohort, a level that is likely to increase as test-and-treat strategies are implemented throughout Latin America. Although room for improvement remains, this finding indicates a fairly strong commitment of cART resources across transmission categories. Even so, programmes that address psychosocial stressors and treat substance use disorders could be invaluable in promoting improved cART use and adherence over time [25,26]. This would, in turn, lead to greater gains in the final care continuum outcome of VS.

Finally, for VS itself, younger age was associated with a reduced probability of suppression. This finding tracks well with results found in cohorts from upper-income countries, including recent work that examined similarities among both behaviourally and perinatally infected youth [24–27]. This result lends further weight to the conviction that public health interventions aiming to link individuals to care, including more aggressive efforts to retain and treat them, may be more cost-effective in immediate health benefits gained and infections averted (through achievement of VS) if younger individuals are prioritized in outreach. A renewed focus on improved social support, access to testing, facilitated healthcare navigation or case management, and a reduction in psychosocial stressors among younger individuals is not meant to detract from resources required to address care for an aging population with HIV infection. However, in efforts to reduce new HIV transmissions and related comorbidities, it may be that wise resource allocation demands more aggressive targeting of younger individuals to blunt the population VL and maintain continuity of care.

Additionally, socially vulnerable or stigmatized groups such as MSM may need special attention to address health inequities across the care continuum, evidenced by reduced likelihood of cART receipt in this population [28,29]. The sustenance of the progress observed in this cohort will depend on expanding the reach of cART programmes, the dedicated linkage of HIV-diagnosed individuals to high quality care and the improved and broader testing of individuals to ensure diagnosis and care at an early disease stage. The populations among whom these tasks may be most difficult to accomplish are likely those that may remain hidden from the healthcare systems and society at large. The public and private sectors of society must promote social inclusion and commit to the passage, promotion and enforcement of antidiscrimination statutes necessary to nullify the effects of discrimination and achieve equity of care and outcomes.

This analysis had multiple limitations, both structural and statistical. First, if patients left cohort sites and sought care elsewhere, they may have been misclassified with respect to outcome, as outcomes were measured within each contributing clinical site and not across the entire health system of a cohort's parent country. That is, a lack of measured clinic attendance, cART use or VS in a particular year may have resulted from a patient obtaining care from a non-CCASAnet clinical site. This limitation is common among observational cohorts, particularly those not linked to surveillance networks. The limitation may have been partially mitigated by the relative prominence and size of most CCASAnet clinical sites in their respective host cities. Second, because this patient population was engaged in care at CCASAnet clinical sites, this study cohort may not be representative of the HIV-infected population within each of the member countries. This selection of a more engaged patient population may have inflated the proportion of patients who were successfully clinically retained, on cART and virally suppressed compared to what might have been observed had the entirety of the HIV-infected population been accessing care. Third, though all models were adjusted by site to control for potential inter-site differences in clinical practice or in healthcare structures, there may have been residual confounding due to fundamental social, economic or healthcare system differences influencing patients’ experiences of HIV care that affected the measured associations. These differences may have included treatment indications such as CD4 thresholds for cART eligibility (noted above), relative poverty of populations being served (including environmental factors related to poverty, such as violence) and predominance of heterosexual transmission among men at some sites (e.g. Peru) versus others (e.g. Chile). Fourth, as this cohort does not currently distinguish current gender identity from birth sex, we could not disentangle transgender individuals from those with MSM as likely HIV transmission category. This could be problematic, as transgender individuals are at particular risk for poor HIV outcomes and sexual/gender minority discrimination. Finally, as measures for diagnosis and linkage to care were unavailable in this cohort, our findings may not pertain to groups without equivalent access to care or knowledge of their own HIV serostatus.

Despite these potential limitations, the use of data from a large, diverse cohort with broad geographic representation in a region important to the HIV epidemic globally and analysis of the data longitudinally, making use of a great strength of clinical cohorts to track changes in these particular outcomes over time, make this work valuable and highly informative. These analyses demonstrate improved care continuum outcomes spanning a decade, among those receiving care throughout clinical care centres in Latin America. The success of expanded cART treatment programmes, alongside long-established public health systems providing near-universal access to care, cannot be underestimated in helping sustain and improve patient engagement in HIV care. However, clinicians and public health practitioners must remain vigilant and efforts must be made to improve retention, cART use and VS rates, particularly among females, those with IDU and MSM HIV transmission category, and younger individuals. Additional research is needed to strengthen the progress that has been made and improve upon it, by identifying impediments to achieving positive care continuum outcomes and their causes in these settings.

## Supplementary Material

Assessing the HIV Care Continuum in Latin America: progress in clinical retention, cART use and viral suppression
