# Supplementary material for: Assessing the HIV Care Continuum in Latin America: progress in clinical retention, cART use and viral suppression
Source: J Int AIDS Soc. 2016 Apr 8;19(1):20636. doi: 10.7448/IAS.19.1.20636 (PMC4827101; doi:10.7448/IAS.19.1.20636)

Supplementary Figure 1. Attrition from the source population of 22,045 CCASAnet patients in clinical care from 2003-2014 due to various exclusion criteria, resulting in the final study population for three HIV Continuum of Care outcomes: retention, cART use, and viral suppression.

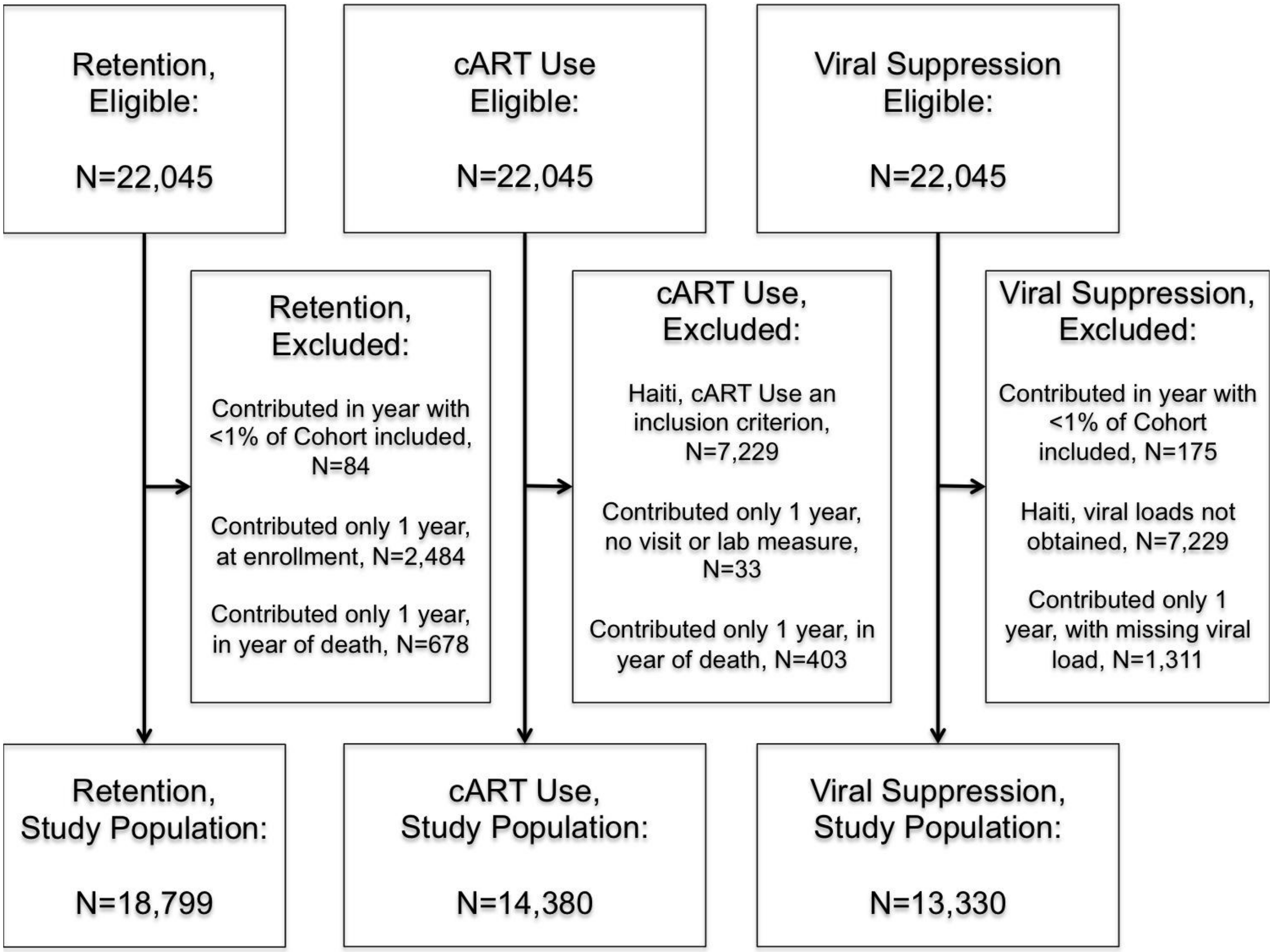

Supplement: Assessing the HIV Care Continuum in Latin America: progress in clinical retention, cART use and viral suppression [file JIAS-19-20636-s001.pdf]
